# Ulcère de jambe récidivant et surinfecté au cours de la maladie de Behçet

**DOI:** 10.11604/pamj.2013.14.139.1785

**Published:** 2013-04-09

**Authors:** Bouomrani Salem, Kilani Ichrak, Nouma Hanène, Chebbi Safouane, Béji Maher

**Affiliations:** 1 Service de Médecine Interne. Hôpital Militaire de Gabes 6000, Tunisie

**Keywords:** Maladie de Behçet, ulcère de jambe, vascularite, Behcet's Disease, leg ulcer, vasculitis

## Abstract

Parmi les manifestations cutanées de la maladie de Behçet, les ulcères de jambes sont exceptionnels mais gardent une implication pronostique fonctionnelle importante surtout dans les formes récidivantes et surinfectées. Nous rapportons l'observation d'un patient âgé de 42 ans porteur de maladie de Behçet depuis l’âge de 27 ans qui fût exploré pour ulcères de jambes bilatéraux, récidivants et invalidants nécessitant l'hospitalisation à plusieurs reprises. A chaque fois les récidives de l'ulcère s'associaient à une poussée cutanéo-muqueuse de la maladie avec notion d'arrêt intempestif de traitement. Il a présenté plusieurs épisodes de surinfection nécessitant le recours aux antibiotiques. Le doppler veineux et artériel ne montraient ni signes d'insuffisance veineuse ni d'artériopathie sous-jacentes. Sous antibiothérapie, en plus des soins locaux et la reprise de la colchicine et des corticostéroïdes, l’évolution se faisait vers la cicatrisation complète des ulcérations mais les récidives étaient fréquentes.

## Introduction

Décrite la première fois en 1937 par le professeur Turque Hulusi Behçet en 1937 [1], la maladie qui porte depuis son nom est une maladie inflammatoire, systémique et chronique d’étiologie inconnue. Elle est plus fréquente chez le sujet jeune, surtout en provenance des pays situés sur l'ancienne « route de soie » [2, 3]. La lésion histologique à l'origine de toutes les manifestations de cette maladie est une vascularite leucocytoclasique touchant l'ensemble des vaisseaux de l'organisme et prédominant sur le réseau veineux [2–4]. Bien que les manifestations cutanées et vasculaires soient fréquentes et bien connues au cours de cette maladie: respectivement 80 et 35-50% [2–4], les ulcères de jambes ne sont pourtant qu'exceptionnellement décrits [2]. Nous en rapportons un cas particulier par son caractère récidivant et ses surinfections fréquentes.

## Patient et observation

M.J Patient Tunisien âgé de 42 ans est connu porteur de la MB depuis l’âge de 27 ans. Le diagnostic était posé devant l'association d'une aphtose buccale récidivante à des aphtes génitaux, des pseudofolliculites nécrotiques, une thrombophlébite du membre inférieur gauche et un Pathergy test positif. Le patient était traité au long cours par colchicine, aspirine et prednisone à faible dose (10 mg/j).

Il présentait des ulcères des deux jambes récidivants et invalidants nécessitant l'hospitalisation à plusieurs reprises. A chaque fois les récidives de l'ulcère s'associaient à une poussée cutanéo-muqueuse de la maladie avec notion d'arrêt intempestif de traitement. L'examen clinique au moment de ces hospitalisations ne trouvait pas de signes de thrombophlébites superficielles au niveau des jambes et les pouls tibiaux postérieurs et pédieux étaient présents. Le doppler veineux des membres inférieurs n'a pas montré de signes d'insuffisance veineuse ni de thrombose profonde concomitante. Le doppler artériel n'a pas montré non plus de signes d'artériopathie sous-jacente. L'indice de pression systolique était à 1,2. La biopsie cutanée réalisée au niveau des berges de l'ulcération montrait des signes de vascularite dermique leucocytoclasique avec des micro-thromboses et une infiltration inflammatoire lymphocytaire du derme profond.

**Figure 1:**
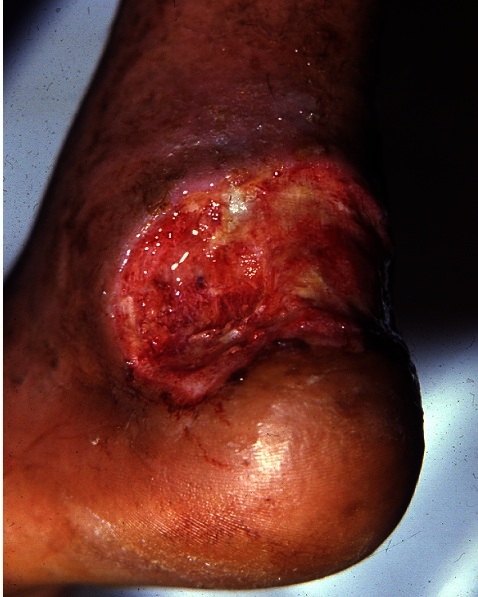
Ulcère rétro bi malléolaire du pied droit propre et bourgeonnant après traitement de la surinfection bactérienne

Le patient avait présenté plusieurs épisodes de surinfection des ulcérations nécessitant le recours aux antibiotiques. La dernière était à pseudomonas aéroginosa (Figure 1). Le diagnostic de ces infections était porté sur un ensemble de signes clinico-biologiques: fièvre septicémique, présence de pus à l'examen local, une hyperleucocytose à polynucléaires neutrophiles et une C-réactive protéine élevée éliminant ainsi une simple colonisation bactérienne d'ulcère.

Avec les traitements locaux, l'antibiothérapie adaptée et la reprise de la colchicine et des glucocorticoïdes l’évolution se faisait vers la cicatrisation complète des ulcérations mais les récidives étaient fréquentes et toujours en rapport avec la mauvaise observance thérapeutique.

## Discussion

Les manifestations cutanées peuvent se voir jusqu’à dans 80% des cas de MB et sont de loin dominées par les ulcérations génitales, les pseudofolliculites nécrotiques, l'hypersensibilité non spécifique aux points de piqûres et/ou d'injections (Pathergy test), l’érythème noueux, les aphtes cutanées et les lésions acnéiformes [5–8]. De même les manifestations vasculaires sont retrouvées dans 35-50% des cas de MB [4]. Le réseau veineux est le plus touché et les atteintes sont volontiers sérieuses et récurrentes [4, 9].

Qu'ils soient considérés comme une atteinte cutanée ou bien vasculaire, les ulcères de jambes restent une manifestation exceptionnelle au cours de cette maladie [5, 6, 10–12]. En effet Vaiopoulis G n'en rapporte qu'un seul cas dans sa série de 202 patients ayant la maladie de Behçet avec manifestations cutanées [6]; soit une fréquence de 0,5%. Kerkeni N. rapporte trois cas dans sa série de 28 cas d'atteintes cutanées au cours de la MB [5] et Takeuchi A. n'a signalé que cinq cas dans sa grande série thérapeutique [11]. Sinon les autres cas sont rapportés de façon sporadique. De notre part nous n'avons observé cette lésion assez particulière qu'une seule fois dans notre série de MB.

Les ulcérations cutanées associées à cette affection peuvent être à type de véritable ulcère chronique de jambe [11] ou bien de petites ulcérations en rapport avec des aphtes cutanés [13] causés par la nécrose des cellules épithéliales [13] ou plus rarement des lésions artérielles et/ou veineuses sous-jacentes [13–15]. Par ailleurs quelques observations de pyoderma gangrenosum ont été rapportées au cours de la MB [4, 13, 16–18].

L'ulcère de jambe au cours de la MB peut simuler un pyoderma gangrenosum et s'associer à une localisation intestinale sévère de la maladie (entéro-Behçet) [18], s'associer à des ulcérations génitales massivement hémorragiques et cacher une thrombose veineuse profonde, en particulier cave inférieure [19] et enfin être bilatéral et récurrent cachant une obstruction de la veine cave inférieure de type « post thrombotique » et incitant donc la réalisation systématique d'une exploration vasculaire cave inférieure [20]. En effet dans sa série de 24 patients ayant la MB avec obstruction cave inférieure, Jackson BT. A trouvé 13 malades soit 54% avec une histoire d'ulcères de jambes récurrents; ces ulcères étaient bilatéraux dans 8 cas sur 13 [20].

Les facteurs intervenant dans la genèse des ulcères cutanés au cours de la MB sont multiples:

**La vascularite sous-jacente:** en effet les vascularites, quelque soit leur type mais surtout nécrosantes, sont reconnues parmi les étiologies rares des ulcères de jambes [13, 21–23]. D'exceptionnels cas d'ulcère de jambe ont été rapportés au cours d'une MB avec vascularite cutanée prouvée histologiquement [14, 15]. Cette vascularite pourrait être leucocytoclasique [14], typique de la MB, ou bien nécrosante lymphohistiocytaire [15] beaucoup moins classique.

**Les thromboses veineuses profondes et les thrombophlébites superficielles:** ces thromboses veineuses prédisposent à l'ulcère cutané de stase et doivent systématiquement faire rechercher une MB [13]. En effet la MB est caractérisé par son tropisme vasculaire et les thromboses veineuses y sont rapportées dans presque le 1/3 des cas [2, 4]. Les membres inférieurs représentent le siège de prédilection de ces thromboses [4]. Dans notre observation on note l'existence dans les antécédents d'un épisode de thrombophlébite de la jambe gauche il y a 10 ans, qui pourrait être pris comme facteur favorisant de l'ulcère cutané au niveau de cette jambe mais qui ne suffit pas à lui seul pour expliquer l'ulcère de la jambe controlatérale.

**Les thérapeutiques utilisées:** en particulier les corticoïdes qui sont à l'origine d'une fragilité cutanée notable [13] et les immunosuppresseurs qui peuvent jouer un rôle dans le déclenchant ou le retard de cicatrisation d'ulcère de jambe [13]. Ces médicaments favorisent aussi la surinfection de ces lésions [13] comme c’était le cas pour notre patient qui était sous corticoïdes au long cours. Notre observation se distingue en plus par le caractère véridique des surinfections; ceci est rare dans la littérature mondiale contrairement à la colonisation qui est classique au niveau des ulcères cutanés.

Dans notre observation, l'absence de signes d'insuffisance veineuse et d'artériopathie sous-jacentes ainsi que l'absence à l'examen clinique et au doppler veineux de signes de thromboses veineuses profondes et/ou superficielles concomitantes aux récidives ulcéreuses et l'existence d'une poussée cutanéo-muqueuse de la maladie à chaque récidive permettent de rattacher directement l'ulcère de jambe à la vascularite de la MB. Les résultats de la biopsie cutanée confirment cette implication. L'antécédent de thrombophlébite du membre inférieur gauche, la corticothérapie au long cours ainsi que l'arrêt intempestif du traitement sont les facteurs favorisants possibles.

Le traitement de l'ulcère de jambe au cours de la MB est souvent difficile à réaliser [11, 24]. Il repose, vu l'existence d'une vascularite sous-jacente, sur la colchicine et la corticothérapie orale à fortes doses [13, 14]. D'autres traitements ont été utilisés avec succès: dapsone et chlorambucil [14, 15]. Les formes récidivantes et résistantes aux thérapeutiques habituelles ont répondu favorablement aux prostaglandines: le OP-1206-CD^®^ qui est un analogue oral actif de la prostaglandine E1 s'est montré très efficace dans le traitement de ces ulcérations avec une régénération observable à partir de la deuxième semaine et une cicatrisation complète au bout d'une année [11].

Actuellement la biothérapie occupe la place de choix dans le traitement de ces atteintes: les anticorps monoclonaux qu'ils soient chimériques (infliximab: Remicade^®^) ou intégralement humains (adalimumab: Humira^®^) se sont montrés efficaces [25]. L'association Humira^®^-methotrexate s'est révélée supérieure à l'infliximab seul [25].

Le traitement de la cause sous-jacente (thrombose veineuse profonde et/ou obstruction cave inférieure) reste le meilleur garant d'une cicatrisation complète et d'absence de récidives ultérieures. Il permet en plus d’éviter des interventions chirurgicales lourdes et inefficaces souvent précipitées pour ces ulcères [20].

## Conclusion

L'ulcère de jambe reste une manifestation exceptionnelle de la maladie de Behçet. Il peut présenter un facteur pronostique, surtout fonctionnel particulièrement dans les formes récidivantes, surinfectées ou résistantes au traitement conventionnel. Un bilan vasculaire se trouve indiqué à la recherche d'une thrombose veineuse profonde ou d'une obstruction cave inférieure sous-jacente. La prise en charge est souvent lourde et multidisciplinaire. La corticothérapie et les anticorps monoclonaux sont à nos jours les thérapeutiques les plus efficaces pour ces lésions.

## References

[1] Behcet H (1937). Uber rezidivierende, aphtose, dürch ein Virus verursachte Geshwure am Munde, am Auge und an den Genitalien. Dermatologische Wochenschrift..

[2] Yurdakul S, Yazici H (2008). Behcet's disease. Best Practice and Research Clinical Rheumatology..

[3] Marshall SE (2004). Behcet's disease. Best Practice and Research Clinical Rheumatology..

[4] Tazi-Mezalek Z, Ammouri W, Maamar M (2009). Les atteintes vasculaires au cours de la maladie de Behçet. Rev Med Interne..

[5] Kerkeni N, Zaraa I, Ayachi J, El Euch D, Mokni M, Ben Osman A (2010). Behçet's disease: A profile of mucocutaneous features. Acta Dermatovenerol Alp Panonica Adriat..

[6] Vaiopoulos G, Konstantopoulou P, Evangelatos N, Kaklamanis PH (2010). The spectrum of mucocutaneous manifestations in Adamantiades-Behçet's disease in Greece. J Eur Acad Dermatol Venereol..

[7] Saadoun D, Wechsler B (2012). Behcet's disease. Orphanet J Rare Dis..

[8] Shang Y, Han SH, Li J, Ren Q, Song F, Chen H (2009). The Clinical Feature of Behçet's Disease in Northeastern China. Yonsei Med J..

[9] Elsharawy MA, Hassan KA, Al-Awami M, Al-Mulhim FA (2004). Dramatic vascular course of Behcet's disease. Saudi Med J..

[10] Jung JY, Kim DY, Bang D (2008). Leg ulcers in Behçet's disease. Br J Dermatol..

[11] Takeuchi A, Hashimoto T (1987). Oral prostaglandin E1 as a therapeutic modality for leg ulcers in Behçet's disease. Int J Clin Pharm Res..

[12] Machtey I (1970). Adverse reaction to penicillamine in a patient with Behcet's syndrome and ulcer of the leg. J Clin Pharmacol New Drugs..

[13] Cacoub P, Francès C, Tazi Z, Delacroix I, Goudeau P (1995). Les ulcères de jambe au cours des maladies systémiques. Rev Med Interne..

[14] Plotkin GR, Patel BR, Shah VN (1985). Behçet's syndrome complicated by cutaneous leukocytic vasculitis: response to prednisone and chlorambucil. Arch Intern Med..

[15] Lee SH, Chung KY, Lee WS, Lee S (1989). Behçet's syndrome associated with bullous necrotizing vasculitis. J Am Acad Dermatol..

[16] Armas JB, Davies J, Davis M, Lovell C, Mc Hugh N (1992). Atypical Behçet's disease with peripheral erosive arthropathy and pyoderma gangrenosum. Clin Exp Dermatol..

[17] Rustin MH, Gilkes JJ, Robinson TW (1990). Pyoderma gangrenosum associated with Behçet's disease: treatment with thalidomide. J Am Acad Dermatol..

[18] Nakamura T, Yagi H, Kurachi K, Suzuki S, Konno H (2006). Intestinal Behcet's disease with pyoderma gangrenosum: a case report. World J Gastroenterol..

[19] Bostankolu A, Aksungur VL, Aksungur EH, Ozpoyraz M, Yücel A, Memisoglu HR (1997). Excessive bleeding from genital ulcers of Behçet's disease. Cutis..

[20] Jackson BT, Thomas ML (1970). Post-thrombotic inferior vena caval obstruction. A review of 24 patients. Br Med J..

[21] (2005). Modules transdisciplinaires. Ulcère de jambe. Ann Dermatol Venereol.

[22] Chen KR, Carlson JA (2008). Clinical approach to cutaneous vasculitis. Am J Clin Dermatol..

[23] Carlson JA, Cavaliere LF, Grant-Kels JM (2006). Cutaneous vasculitis: diagnosis and management. Clin Dermatol..

[24] Liu P (1984). Care of patients with Behcet's syndrome-caused serious ulcer of the legs. Zhonghua Hu Li Za Zhi..

[25] Atzeni F, Leccese P, D'Angelo S, Sarzi-Puttini P, Olivieri I (2010). Successful treatment of leg ulcers in Behçet's disease using adalimumab plus methotrexate after the failure of infliximab. Clin Exp Rheumatol..

